# Case report: An unusual case of desmin myopathy associated with heart failure and arrhythmia

**DOI:** 10.3389/fcvm.2022.944459

**Published:** 2022-07-25

**Authors:** Xuhan Liu, Yuan Liu, Bo Li, Lin Wang, Weihua Zhang

**Affiliations:** Department of Cardiovascular Medicine, The First Hospital of Jilin University, Changchun, China

**Keywords:** heart failure, desmin (DES), arrhythmia, ARNI, cardiac conduction system

## Abstract

**Introduction:**

Desmin myopathy is a novel desmin (DES) indel mutation that causes severe atypical cardiomyopathy as well as atrioventricular block and skeletal myopathy. The mutation of the gene of the nodal tail causes myocardial injury. Rarely does desmin myopathy cause bilateral ventricular changes.

**Case presentation:**

We present a case of a 48-year-old man admitted with dyspnea and edema of both lower extremities. Due to bilateral lower limb weakness and calf muscle atrophy, gene sequencing was performed. The results showed that there was a pure missense mutation in the 8th exon region of the DES gene (c.1366G>A), encoding amino acid p.G456R (glycine>arginine). Supplementary examination suggests a high possibility of heart failure, atrial flutter, and desmin myopathy. Atrial flutter was treated by radiofrequency ablation. The clinical symptoms were stable after oral administration of rivaroxaban, coenzyme Q10, and ARNI.

**Conclusion:**

In our case, mutation results are the gold standard for the diagnosis of desmin myopathy. Cardiac magnetic resonance can define the extent and degree of cardiomyopathy and quantitatively evaluate cardiac function. At present, there is a lack of specific treatment for proteolytic myopathy. Therefore, the treatment for heart failure proves effective. Due to the multiple systems involved, early diagnosis and multidisciplinary management are critical to improving patient outcomes.

## Keypoints

- Desmin myopathy is a novel desmin (DES) indel mutation. The condition is characterized by severe atypical cardiomyopathy combined with atrioventricular block and skeletal myopathy.
- Bilateral ventricular changes due to desmin myopathy are rare.
- Currently, there is a lack of specific treatments for desmin myopathy. But treatment regimens that correct arrhythmias and improve heart failure and myocardial metabolism have proven effective.

## Introduction

Desmin myopathy is a subtype of myofibromyopathy in which the DES gene, the pathogenic gene, is located on chromosome 2q35 and contains nine exons (1). The age of onset of desmin myopathy is from 2 to 48 years old. Both men and women can be affected, with the autosomal dominant inheritance of family cases (2). Heart failure, severe arrhythmias, and respiratory muscle involvement with respiratory failure caused by cardiac damage in desmin myopathy are direct factors for poor prognosis. This paper shows a case of a man admitted with dyspnea and bilateral lower limb as the first symptoms of a severe desmin myopathy complicated by arrhythmia.

## Case report

We present a case of a 41-year-old man who was admitted to the First Hospital of Jilin University on June 11, 2020, with intermittent dyspnea for 8 years and bilateral lower limb edema for 2 years, aggravated for 3 months. He had been suffering from bilateral lower limb weakness with calf muscle atrophy for 30 years, and electromyography suggested myogenic damage, and muscle biopsy showed myotonic changes. The results of sequencing suggested a pure missense mutation (c.1366G>A) in the exon eight regions of the desmin (Desmin, *DES*) gene, encoding amino acid p.G456R (glycine>arginine).The patient's muscle biopsy was performed with immunohistochemical staining. Muscle fibers were not stained by Desmin staining. Consider the possibility of desmin myopathy, only whole-exon DES testing was performed. The parents of the preexisting patient were non-consanguineous, both carriers of the heterozygous variant at the locus, and had no clinical manifestations such as myasthenia gravis. The parents were tested at the same time.

**Figure 1:**
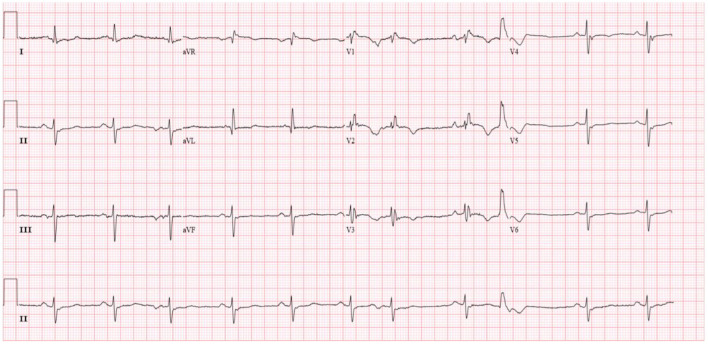
Patient's electrocardiogram (sinus rhythm, heart rate 66 beats/min, incomplete right bundle branch block, left anterior branch block, premature atrial, and ventricular beats).

**Figure 2:**
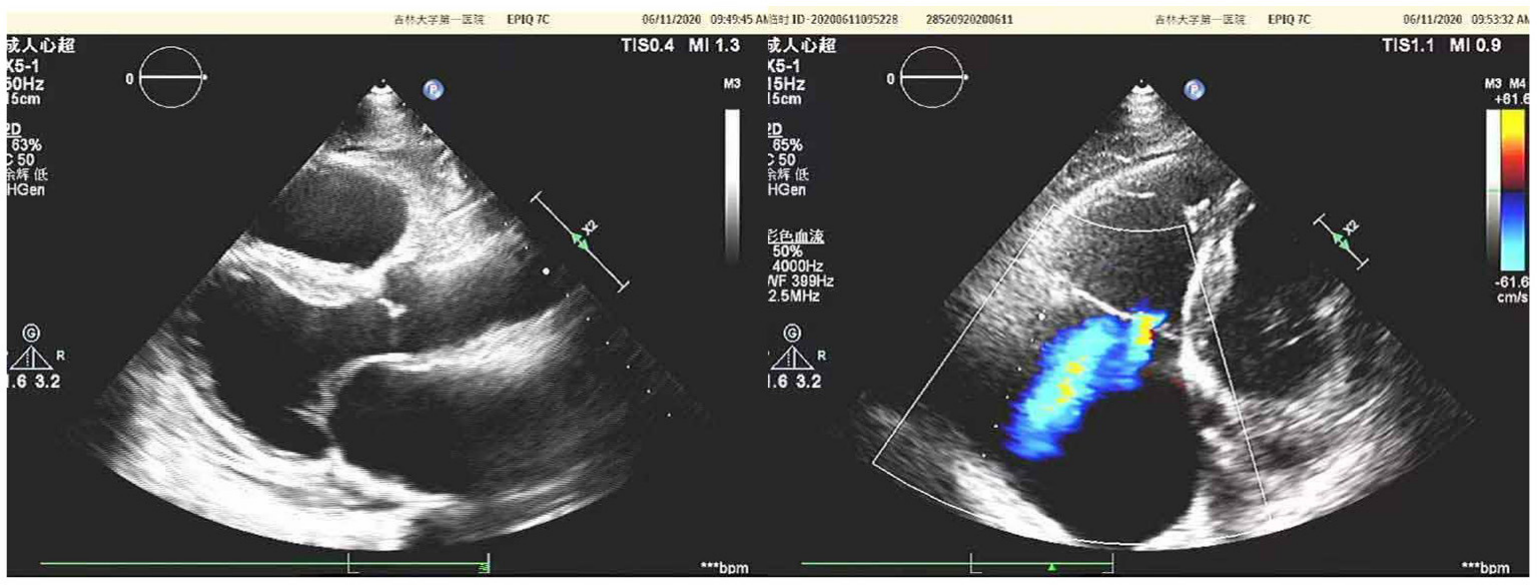
Echocardiographic of the patient's heart [a left ventricular ejection fraction (LVEF) of 46% (M-type Teichholz method), a right ventricular internal diameter of 26 mm, a right atrial diameter of 54 × 64 mm, diffusely attenuated right ventricular wall pulsation, and the tricuspid plane systolic excursion (TAPSE) was 12 mm, and the tricuspid regurgitation was severe].

Diuretics and multifunctional monitoring were promptly administered. Laboratory findings are as followed: serum creatine kinase (CK) 223 U/L. N terminal-pro B type natriuretic peptide (NT-proBNP) 1,770 pg/mL. cTnI (cardiac troponin I) was in the normal range. Blood gas analysis showed: partial pressure of oxygen of 89 mmHg (1 mmHg = 0.133 kPa) and partial pressure of carbon dioxide of 34 mmHg. Electrocardiogram (ECG) showed: sinus rhythm, incomplete right bundle branch block, left anterior branch block, atrial premature beat, and ventricular premature beat (Figure 1). Radionuclide lung ventilation-perfusion imaging was not abnormal. Pulmonary function tests showed that exertional spirometry was 48% of the expected value, suggesting restrictive ventilatory dysfunction. The echocardiogram showed a left ventricular ejection fraction (LVEF) of 46% (M-type Teichholz method), a right ventricular internal diameter of 26 mm, a right atrial diameter of 54 × 64 mm, diffusely attenuated right ventricular wall pulsation, and the tricuspid plane systolic excursion (TAPSE) was 12 mm, and the tricuspid regurgitation was severe (Figure 2).

**Figure 3:**
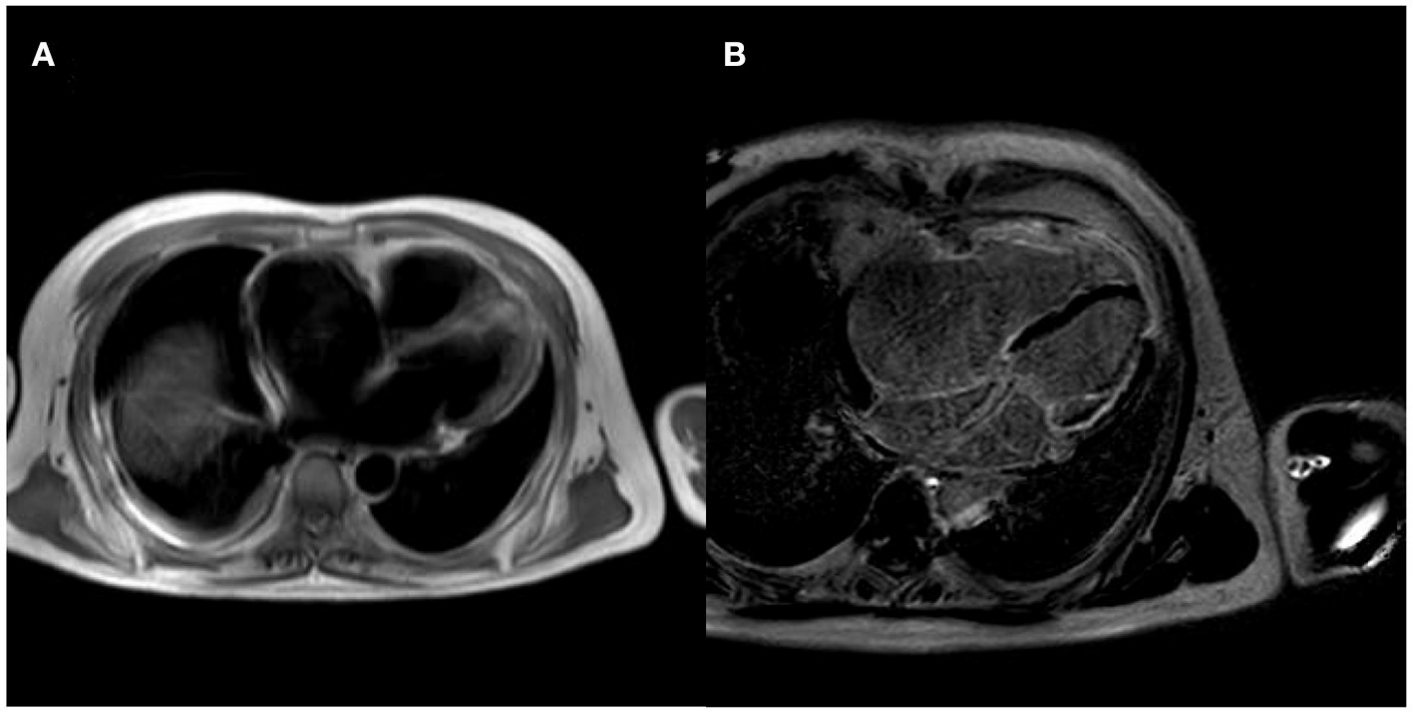
MRI of the patient's heart [**(A)** is a whole heart cross-sectional view, which shows a small left atrium and an enlarged right atrium; **(B)** is a delayed scan, which shows diffuse strip enhancement of the left ventricular lateral wall, apical region, and right ventricular wall, with some transmural-like enhancement].

After controlling the patient's heart rate with medication, a cardiac magnetic resonance examination was performed. The result of magnetic resonance imaging (MRI) (Figure 3) showed that the left atrial and left ventricular internal diameters were normal, the right atrium was 61 mm anterior-posterior, and the right ventricular transverse diameter was 43 mm. The delayed scan showed diffuse striated enhancement of the left ventricular lateral wall, apex of heart, and right ventricular wall, with some transmural enhancement, LVEF 35%, and cardiac output 2.61 L/min.

In this patient, the diagnosis was clear: desmin myopathy caused myocardial injury, right heart dilatation, total heart failure, cardiac function class 3 (NYHA class), arrhythmia-RBBB, LAFB, atrial premature beat, ventricular premature beat. The patient was treated with a diuretic treatment (Furosemide, Spironolactone orally), cardiac stimulant (digoxin orally), and sacubitril/valsartan 50 mg 2 times/d orally, which relieved dyspnea, nocturnal paroxysmal dyspnea, and bilateral lower limb edema. Two months later, the patient's reexamination showed improvement: NT-proBNP 1,090 pg/mL.

**Figure 4:**
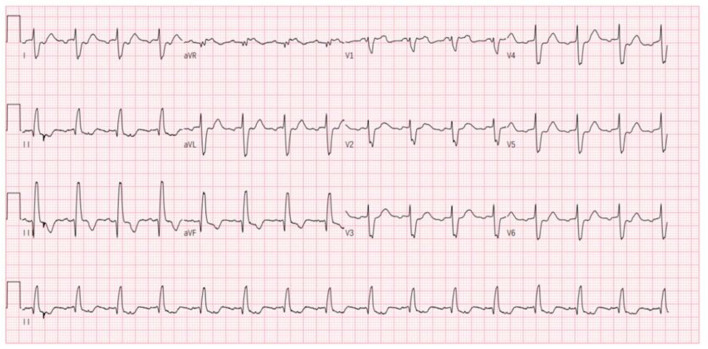
Patient's ECG after this admission (ectopic rhythm, heart rate 93 beats/min, paroxysmal atrial flutter 2:1 conduction, non-specific intraventricular conduction block, prolonged QT interval).

Nevertheless, the patient was admitted to our hospital again on December 18, 2020, with palpitations for 1 day. Physical examination: pulse 98 times/min, respiration 19 times/min, blood pressure 112/70 mmHg. There is no distension of jugular veins, coarse breath sounds in both lungs, and no rales were heard. The heart rate was 98 beats/min with arrhythmias. A grade 2/6 systolic murmur could be heard under the xiphoid. The gastrocnemius muscles were atrophied bilaterally. Muscle strength examination: grade IV in the distal part of both lower limbs, grade II in the dorsal extension of both feet. Supplementary examination: B type natriuretic peptide (BNP) 934 pg/mL; digoxin concentration: 0.43 ng/mL; serum ion and cTnI levels normal range. The patient did not have an ECG at the time of the palpitations attack. Still, the ECG after admission showed ectopic rhythm, paroxysmal atrial flutter (2:1 conduction), non-specific intraventricular block, and prolonged QT interval (Figure 4). The echocardiogram showed an LVEF of 29% (M-type Teichholz method), an anterior-posterior right ventricular diameter of 34 mm, a right atrial diameter of 55 × 68 mm, and a TAPSE of 8 mm, a decreased right ventricular function, and moderate tricuspid regurgitation. The diagnosis of atrial flutter was clear, and the patient underwent cardiac radiofrequency ablation with temporary pacemaker implantation on the 5th day of admission. Preoperative esophageal echocardiography and left atrial computed tomographic angiography (CTA) did not show any left atrial or left atrial appendage thrombus.

**Figure 5:**
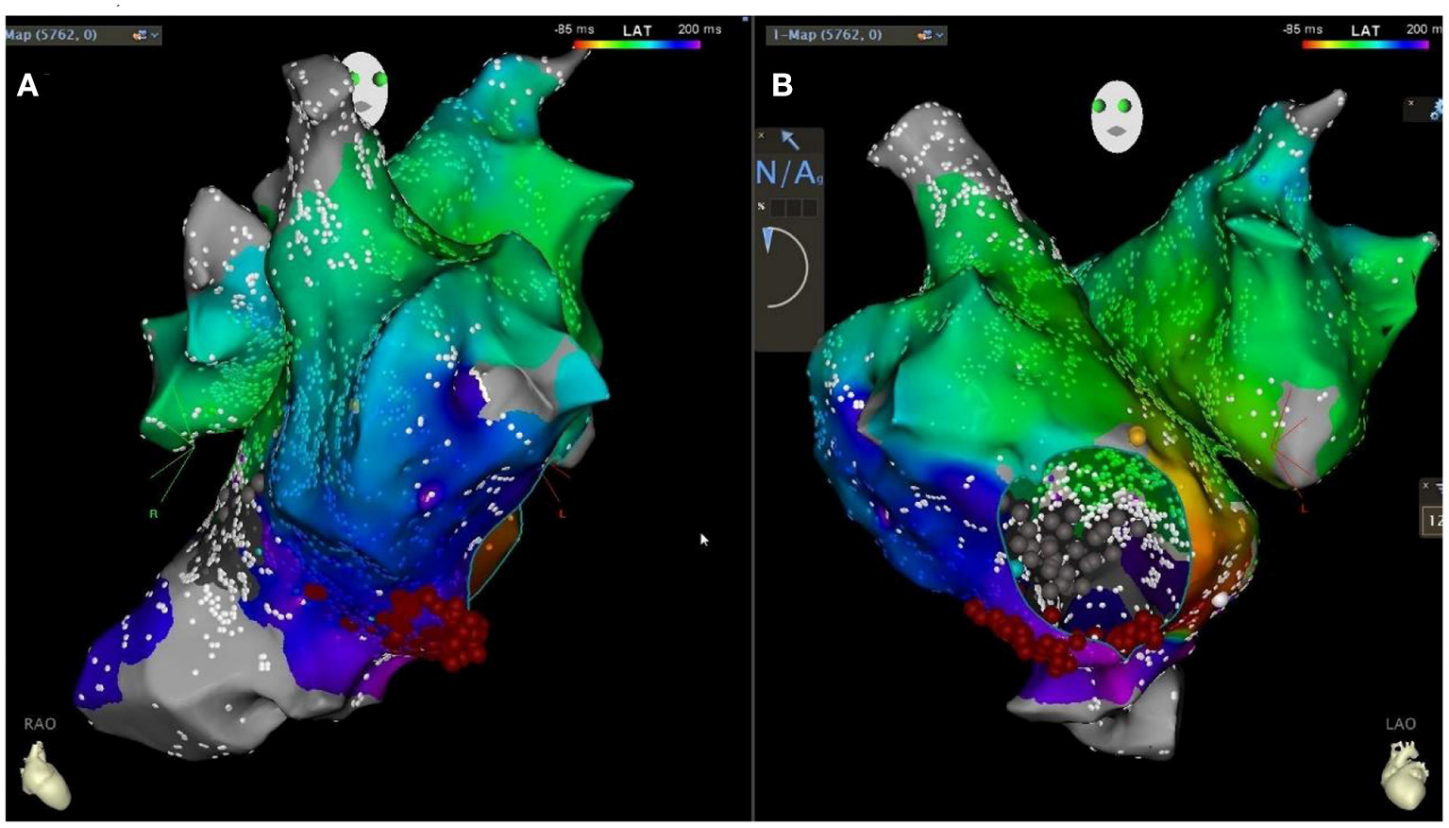
Patient's CARTO downstream agitation specimen [retrograde bell-like folding atrial flutter around the tricuspid annulus, tachycardia circumference 300ms, linear ablation along the tricuspid isthmus in the right anterior oblique 30 degrees in **(A)** and left anterior oblique 45 degrees in **(B)** position].

**Figure 6:**
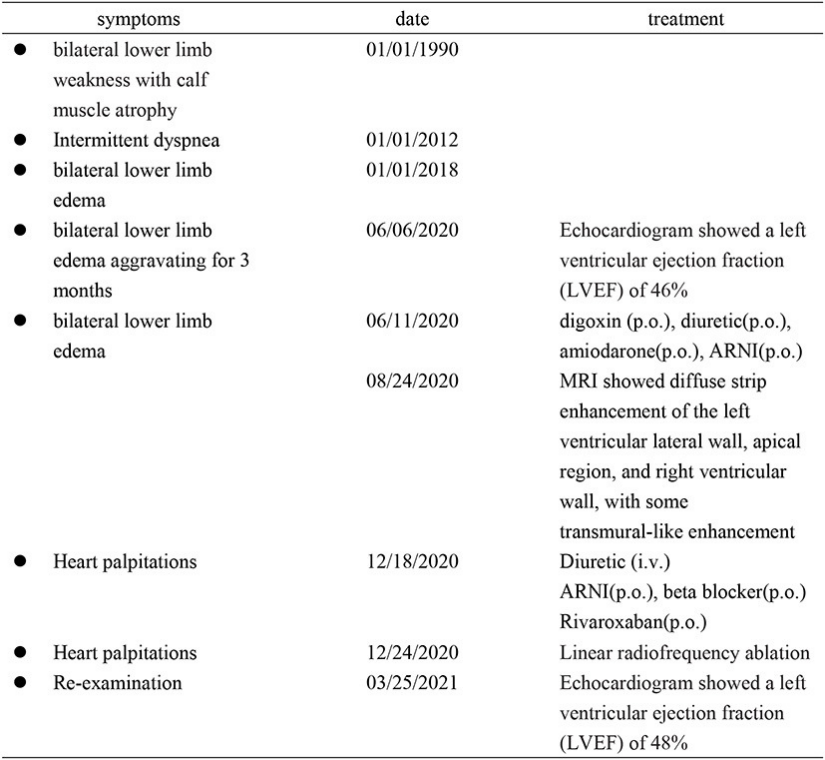
The patient's timeline of admission, diagnosis, and treatment.

To correct the arrhythmia, a star mapping electrode was applied, and the result was tricuspid isthmus reverse clock reentry atrial flutter. Linear radiofrequency ablation was performed along the tricuspid isthmus (Figure 5), which lead to atrial flutter terminating and recovering sinus rhythm, with intermittent borderline rhythm. After giving temporary cardiac pacing, the postoperative HV interval was 69 ms. The patient's postoperative ECG showed sinus rhythm with non-specific intraventricular block and prolonged QT interval. The temporary pacemaker was removed on the second postoperative day. After the discharge, the patient was given a diuretic (furosemide orally), anticoagulation (rivaroxaban orally), and coenzyme Q10 therapy. Due to low blood pressure, the dose of sacubitril/valsartan was reduced to 25 mg orally twice. The patient was followed up regularly (Figure 6).

## Discussions

Desmin myopathy is a subtype of myogenic fibromyopathy with a causative gene, the *DES* gene, located on chromosome 2q35 and containing nine exons (1). The age of onset of desmin myopathy is 2–48 years, both sexes can be affected, and family cases are mainly autosomal dominant (2). In this case, both parents are carriers of the mutated gene. Neither of them has developed the disease; the preexisting patient is a homozygous, consistent with autosomal recessive inheritance. The disease mainly presents with symmetrical distal muscle weakness of the lower extremities, in some cases involving the myocardium and cardiac conduction system (1). A meta-analysis by Carvalho et al. showed that ~15–50% of patients have restrictive respiratory insufficiency (3, 4). Van Spaendonck-Zwarts et al. (5) showed that most patients with DES mutations present with combined skeletal and cardiac myopathy, with ~75% of patients with DES mutations presenting with cardiac symptoms, of which only 22% have an isolated cardiac phenotype (6). In this case, the patient's first symptom was myasthenia with myasthenia gravis, elevated serum CK, and electromyography, and muscle biopsy pathology confirmed the presence of myopathy, consistent with combined skeletal and cardiac diseases, and with pulmonary function suggesting restrictive respiratory insufficiency. It has been reported that the junctional protein tail is involved in the regulation of intermediate filament dynamics, and its mutation mainly causes myocardial damage. In our patient, the *DES* gene mutation site was in the region encoding the structure of the junctional protein tail (c.1366G>A), resulting in the substitution of glycine by arginine, leading to a conformational change in this structural domain and increased sensitivity of myocytes to mechanical forces associated with contraction, triggering contractile dysfunction in cardiac, and skeletal muscle and promoting myocardial remodeling.

Echocardiography and cardiac MRI can clarify the extent and degree of myocardial disease and quantitatively assess cardiac function. Desmin myopathy often presents as different types of cardiomyopathy, including dilated cardiomyopathy, hypertrophic cardiomyopathy, restrictive cardiomyopathy, arrhythmogenic right ventricular cardiomyopathy, and non-dense cardiomyopathy (7, 8). Arrhythmias include atrioventricular block, bundle branch block, atrial fibrillation, and ventricular tachycardia (9). CMR can differentiate between injured and normal myocardium. T2-weighted imaging sequences of myocardial hyper-signal reflect myocardial edema, and perfusion sequences can detect myocardial ischemia (10). The echocardiogram showed severe right heart damage, consistent with restrictive cardiomyopathy. Still, the cardiac MRI showed that the left ventricular myocardium was also involved, and the LVEF value was significantly reduced. In this case, the right atrium was dilated, resulting in right atrial remodeling, which facilitated the formation of a foldback loop, resulting in rapid atrial pulsation, and eventually atrial flutter. On the contrary, the loss of atrial pump function and the desynchronization of atrial and ventricular motion during atrial flutter can further induce and aggravate heart failure, creating a vicious circle between the two, and significantly reducing activity tolerance. In addition, junctional proteins are also expressed in Purkinje fibers and intercalated discs; therefore, junctional protein myopathy often causes conduction block arrhythmias (11). In this case, the electrocardiogram shows a non-specific intraventricular block, suggesting an involvement of the cardiac conduction system. If the lesion worsens, a complete AV block at the branch level may occur, leading to syncope or sudden cardiac death.

In this case, the patient underwent linear ablation at the isthmus of tricuspid valve. During the ablation process, atrial flutter was terminated and sinus rhythm was restored, and borderline rhythm occurred intermittently. Temporary cardiac pacing was performed to verify the integrity of the ablation line. Low right atrium and coronary sinus pacing, PPI measurement of 200 ms, indicating tricuspid isthmus bidirectional block, indicating a successful operation. The amount of radiation in radiofrequency ablation surgery is used on-demand. In this radiofrequency ablation operation, we have tried to reduce the radiation dose of the patient, and the radiation dose of this patient is about 30–40 MGy. In addition to determining potential clinical benefits, zero X-ray also defines safe technical advantages in terms of lower ionizing radiation exposure. In surgery for the future, we should try to less rays, achieve zero ray of surgery, reduce the damage to the body (12).

There is a lack of specific treatment for myocardial damage in junctional protein myopathy. Medicine can only be based on the guidelines for heart failure (13). In patients with heart failure combined with atrial flutter, aggressive radiofrequency ablation is given to treat the atrial flutter and synchronize the atrial motion, which is beneficial to improving the prognosis of patients with heart failure. The patient, in this case, applied cardiotonic and diuretic drugs but not β-blockers, considering that their adverse inotropic effects may aggravate right heart failure. In this case, the patient's symptoms were relieved after applying anti-heart failure drugs, and the NT-proBNP level was reduced. However, the echocardiogram after the onset of atrial flutter indicated that the cardiac function was lower than before, suggesting that the atrioventricular desynchronization caused by atrial flutter may reduce the efficacy of anti-cardiac failure drugs. The long-term effectiveness of radiofrequency ablation in patients with heart failure combined with atrial flutter needs to be further observed. Heart transplantation may be considered in patients with advanced chronic heart failure (2). However, respiratory muscle damage due to myopathy can still lead to death, so the benefit of heart transplantation may be limited. Because of the abnormalities in mitochondrial function in Desmin myopathy, the administration of drugs such as coenzyme Q10 and trimetazidine to improve energy metabolism may be beneficial (14). Due to diffuse cardiac conduction system lesions, guidelines recommend implanting a permanent pacemaker if the patient develops severe bradyarrhythmias (15). As a result of gaining experience in developing muscle-specific synthetic promoters, scientists can develop constructs that mimic the distinctive expression profile of muscles-specific proteins and fully recover their lost functions (16). For those with inherited muscle metabolic diseases, this treatment may be particularly useful.

Heart failure due to myocardial damage in desmin myopathy, severe arrhythmias, and respiratory muscle involvement complicated by respiratory failure are direct factors in the poor prognosis of the patient. In this case, the patient presented with total heart failure, bundle branch block, atrial flutter, and restrictive ventilation dysfunction, suggesting the presence of respiratory muscle involvement, presumably with a poor long-term prognosis. CMR is an essential imaging test for detecting early cardiac involvement in desmin myopathy and predicting patient prognosis; therefore, early completion of CMR in patients with suspected desmin myopathy should be recommended to achieve earlier diagnosis, early treatment, reduce further myocardial injury, and improve patient survival. Most junctional protein myopathies are missense mutations or small deletion mutations, and in this case, the patient had a missense mutation. Since junctional proteins are scaffolding proteins that connect organelles, there are various secondary and tertiary molecular and cytopathological mechanisms *in vitro* and *in vivo* that affect different cellular compartments. There is no specific therapy yet. It is hoped that in the future new advances in molecular and cellular biology will allow the development of molecular treatments applied to patients with junctional protein myopathy.

## Data availability statement

The original contributions presented in the study are included in the article/supplementary materials, further inquiries can be directed to the corresponding author.

## Ethics statement

Written informed consent was obtained from the individual(s) for the publication of any potentially identifiable images or data included in this article.

## Author contributions

XL, YL, BL, LW, and WZ: substantial contributions to the conception or design of the work, or the acquisition, analysis, or interpretation of data for the work, drafting the work or revising it critically for important intellectual content, final approval of the version to be published, and agreement to be accountable for all aspects of the work in ensuring that questions related to the accuracy or integrity of any part of the work are appropriately investigated and resolved. YL: writing—review & editing. BL: conceptualization, methodology, and visualization. LW: investigation, formal analysis, and writing—review & editing. All authors contributed to the article and approved the submitted version.

## Funding

This work was supported by the Heart failure cohort established (2016YFC1301002).

## Conflict of interest

The authors declare that the research was conducted in the absence of any commercial or financial relationships that could be construed as a potential conflict of interest.

## Publisher's note

All claims expressed in this article are solely those of the authors and do not necessarily represent those of their affiliated organizations, or those of the publisher, the editors and the reviewers. Any product that may be evaluated in this article, or claim that may be made by its manufacturer, is not guaranteed or endorsed by the publisher.
