# Corrigendum: Case report: An unusual case of desmin myopathy associated with heart failure and arrhythmia

**DOI:** 10.3389/fcvm.2022.1002478

**Published:** 2022-10-03

**Authors:** Xuhan Liu, Yuan Liu, Bo Li, Lin Wang, Weihua Zhang

**Affiliations:** Department of Cardiovascular Medicine, The First Hospital of Jilin University, Changchun, China

**Keywords:** heart failure, desmin (DES), arrhythmia, ARNI, cardiac conduction system

In the published article, there were some errors.

A correction has been made to **Abstract**, *Conclusion*, Paragraph 1. This sentence previously stated “In our case, mutation results are the gold standard for the diagnosis of nodular myopathy.” The corrected sentence is “In our case, mutation results are the gold standard for the diagnosis of desmin myopathy.”

A correction has been made to **Introduction**, Paragraph 1. This sentence previously stated “…and respiratory muscle involvement with respiratory failure caused by cardiac damage in nodular myopathy are direct factors for poor prognosis.” The corrected sentence is “…and respiratory muscle involvement with respiratory failure caused by cardiac damage in desmin myopathy are direct factors for poor prognosis”.

A correction has been made to **Case report**, Paragraph 4. This sentence previously stated “So far, the patient's clinical diagnosis is clear, which is myocardial damage in desmin myopathy.” The corrected sentence is “In this patient, the diagnosis was clear: desmin myopathy caused myocardial injury”.

The authors apologize for these errors and state that this does not change the scientific conclusions of the article in any way. The original article has been updated.

## Publisher's note

All claims expressed in this article are solely those of the authors and do not necessarily represent those of their affiliated organizations, or those of the publisher, the editors and the reviewers. Any product that may be evaluated in this article, or claim that may be made by its manufacturer, is not guaranteed or endorsed by the publisher.

